# Leveraging Tweets for Artificial Intelligence Driven Sentiment Analysis on the COVID-19 Pandemic

**DOI:** 10.3390/healthcare10050910

**Published:** 2022-05-13

**Authors:** Nora A. Alkhaldi, Yousef Asiri, Aisha M. Mashraqi, Hanan T. Halawani, Sayed Abdel-Khalek, Romany F. Mansour

**Affiliations:** 1 Department of Computer Science, College of Computer Sciences and Information Technology, King Faisal University, Al-Ahsa 31982, Saudi Arabia; nalkhaldi@kfu.edu.sa; 2 Department of Computer Science, College of Computer Science and Information Systems, Najran Univesity, Najran 61441, Saudi Arabia; yasiri@nu.edu.sa (Y.A.); ammashraqi@nu.edu.sa (A.M.M.); 3 Department of Mathematics, College of Science, Taif University, Taif 21944, Saudi Arabia; sabtalb@tu.edu.sa; 4 Department of Mathematics, Faculty of Science, New Valley University, El-Kharga 72511, Egypt; romanyf@sci.nvu.edu.eg

**Keywords:** COVID-19, sentiment analysis, Twitter, mental illness, deep learning, natural language processing

## Abstract

The COVID-19 pandemic has been a disastrous event that has elevated several psychological issues such as depression given abrupt social changes and lack of employment. At the same time, social scientists and psychologists have gained significant interest in understanding the way people express emotions and sentiments at the time of pandemics. During the rise in COVID-19 cases with stricter lockdowns, people expressed their sentiments on social media. This offers a deep understanding of human psychology during catastrophic events. By exploiting user-generated content on social media such as Twitter, people’s thoughts and sentiments can be examined, which aids in introducing health intervention policies and awareness campaigns. The recent developments of natural language processing (NLP) and deep learning (DL) models have exposed noteworthy performance in sentiment analysis. With this in mind, this paper presents a new sunflower optimization with deep-learning-driven sentiment analysis and classification (SFODLD-SAC) on COVID-19 tweets. The presented SFODLD-SAC model focuses on the identification of people’s sentiments during the COVID-19 pandemic. To accomplish this, the SFODLD-SAC model initially preprocesses the tweets in distinct ways such as stemming, removal of stopwords, usernames, link punctuations, and numerals. In addition, the TF-IDF model is applied for the useful extraction of features from the preprocessed data. Moreover, the cascaded recurrent neural network (CRNN) model is employed to analyze and classify sentiments. Finally, the SFO algorithm is utilized to optimally adjust the hyperparameters involved in the CRNN model. The design of the SFODLD-SAC technique with the inclusion of an SFO algorithm-based hyperparameter optimizer for analyzing people’s sentiments on COVID-19 shows the novelty of this study. The simulation analysis of the SFODLD-SAC model is performed using a benchmark dataset from the Kaggle repository. Extensive, comparative results report the promising performance of the SFODLD-SAC model over recent state-of-the-art models with maximum accuracy of 99.65%.

## 1. Introduction

COVID-19 is a communicable disease that can be transferred or spread mainly by the tiny droplets released by the individual during sneezing, coughing, and also while talking. It is currently becoming a source of anxiety depression and stress, owing to the false information that is to be posted on social media. The mental well-being of people is severely affected due to the fast spread of incorrect information on social media [1,2]. Due to the present situation of lockdown and social distancing, people are mainly dependent on, or even addicted to, the internet and mobile phones, as revealed by reports indicating that the highest number of activities are performed [1] on social media. At the time of lockdown, traffic on social media has extremely increased [3]. Among all other social media, Twitter ranks first in spreading COVID news [4,5]. The devastating part of such news is subjective because it involves mostly personal thoughts and confusion, which leads to intentional fake information, negativity, and uncertainty in the human community [6]. Meanwhile, this condition is seeking the interest of researcher scholars to make calculable analyses to create a wholesome picture. This study mainly aims at sentiment analysis based on Twitter datasets with regard to COVID-19 through a supervised machine learning algorithm.

During lockdowns, all individuals, particularly teenagers, usually spend more time on Twitter, and in fact, users are more active than at any other time. The reason behind this is to receive up-to-date information regarding COVID-19 news. Meanwhile, they share their thoughts and feelings with friends and society through a medium. Therefore, in this pandemic situation, the analysis of Twitter data has received attention from the research community. Sentiment analysis (SA) is a technical study that deals with the opinions, attitudes, and emotions of people [7]. It is considered an efficient way to calculate people’s opinions on specific topics. Additionally, SA is able to convey several impacts on the community in various means. Additionally, SA summarizes the different anxieties and mental health conditions of people that arise during the pandemic situation. We can quickly identify the depression status and panic disorder of individuals in a community from the SA outcome [8]. The only solution to bring positivity to society is to apply various virtual depression optimizers for that depressed person. It should be mentioned that the success of most applications is based on the sentiments of social users. SA for active users is considered one of the efficient ways of tracking public opinion. In this pandemic situation, these kinds of studies have made important contributions to helping policymakers and governments.

Based on this background, this paper presents a new sunflower optimization with deep-learning-driven sentiment analysis and classification (SFODLD-SAC) on COVID-19 tweets. The presented SFODLD-SAC model initially preprocesses the tweets in distinct ways such as stemming, removal of stopwords, usernames, link punctuations, and numerals. In addition, the TF-IDF model is applied for the useful extraction of features from the preprocessed data. Moreover, a cascaded recurrent neural network (CRNN) model is employed to analyze and classify sentiments. Finally, the SFO algorithm is utilized to optimally adjust the hyperparameters involved in the CRNN model. The simulation analysis of the SFODLD-SAC model is performed using a benchmark dataset from the Kaggle repository. In short, the paper’s contributions are as follows:

- An intelligent SFODLD-SAC model is presented consisting of TF-IDF-based feature extraction, CRNN classification, and SFO-based hyperparameter optimization for COVID-19 tweet analysis. To the best of our knowledge, the SFODLD-SAC model has been never presented in the literature;
- The SFODLD-SAC technique involves the design of an SFO algorithm to optimally choose the hyperparameters, which helps in increasing the classification accuracy and avoids computational overhead;
- The performance of the SFODLD-SAC model is validated using a benchmark dataset from the Kaggle repository, and the results are investigated under distinct sizes of training/testing data.

The rest of this paper is organized as follows: Section 2 offers related research, and Section 3 discusses the proposed model. Then, Section 4 elaborates on the experimental validation with the benchmark Kaggle dataset, and Section 5 draws the conclusions of the paper.

## 2. Literature Review

This section offers a detailed review of existing SA models related to COVID-19. Researchers in [9] analyzed Indian people’s sentiment during the lockdown. They used some popular hashtags for measuring negativity and positivity in people. Samuel et al. [10] highlighted public sentiments related to the COVID-19 pandemic using two machine learning (ML) classification techniques. The researchers in [11] presented an architecture, in which a deep-learning-based language model was applied through long short-term memory (LSTM) recurrent neural network for sentimental analysis during the increase in COVID-19 cases in India. In [12], bidirectional encoder representation conducted COVID-19 tweet data analysis from a Transformer-based (BERT) model. Gulati et al. [13] implemented a comparative analysis of an ML-based classifier. This classifier was employed for above 72,000 tweets related to COVID-19. Mujahid et al. [14] employed a Twitter dataset comprising 17,155 tweets regarding e-learning. ML and DL methods showed the potential, suitability, and capability for object detection, natural language processing, and image processing tasks. Luo and Xu [15] presented a DL method to explore customer opinion regarding restaurant features and to discover reviews with mismatched ratings. This study strengthens the extant literature by analyzing restaurant reviews posted during the COVID-19 pandemic and finding a DL algorithm for text mining tasks [16].

Singh et al. [17] proposed a DL technique for SA of Twitter statistics based on COVID-19 analyses. The suggested model depends on the LSTM–RNN-based network and improved featured weight by attention layer. This approach makes use of an improved feature transformation architecture through the attention model. Yin et al. [18] conducted a study based on COVID-19 vaccination on Twitter. The authors analyzed the deliberations of individuals in terms of this research topic and the emotional polarization between vaccine brands and perceptions of countries. The results showed that the majority of individuals trust the usefulness of vaccines, and they are ready to vaccinate themselves. In another study [19], the authors focused on increasing the consideration of public awareness of the COVID-19 pandemic trend and uncovering meaningful themes of concern posted by Twitter users in the English language. An NLP method and the latent Dirichlet allocation model was utilized to classify cluster and identify themes based on keyword analysis, along with identifying the most common twitter topics. In [20], data from the Arabic COVID-19-based tweet dataset were gathered. The data were processed according to the ML prediction model. The results showed that applying the SVM classification together with bigram in TF-IDF outperformed other algorithms, with 85% accuracy.

Lyu et al. [21] identified sentiments and topics in COVID-19 vaccine-interrelated conversation among the public on social networking platforms and discriminate the relevant modifications in sentiments and topics over time for a good understanding of public emotions, perceptions, and concerns that might affect the accomplishment of herd immunity objectives. Basiri et al. [22] presented a methodology according to the fusion of four DL and one traditional supervised ML method for SA of COVID-based twitters from eight countries. Moreover, the authors analyzed COVID-based searches using Google Trends for a good understanding of the changes in sentimental patterns at dissimilar places and times. Imran et al. [23] analyzed the reaction of citizens from various cultures to the novel COVID-19 and people’s sentiments regarding subsequent actions taken by many countries. The deep LSTM model was utilized for assessing the emotions and sentimental polarities from extracted tweets. In [24], GloVe and fastText were tested as word embedding. Data collected from Twitter were prepared as stemmed and unstemmed datasets.

In short, SA can be considered a meaningful source of data mining, particularly for circumstances relevant to the requirement of examining massive quantities of publicly relevant data, such as investigating public behavior concerning the COVID-19 pandemic and its outcome on people’s lives. Furthermore, it is desirable to improve decision makers’ countermeasures and offer them an effortless method with a collection of common rules that assist complex decision-making processes depending on people’s sentiments and via examining and sorting an essential set of key features for COVID-19 posts. Thus, the proposed study in this paper varies from earlier research in combining DSS with SA for improving government decisions at the time of COVID-19. The use of the SFODLD-SAC model offers more insights and achieves better performance than other state-of-the-art techniques.

## 3. Materials and Methods

**Figure 1:**
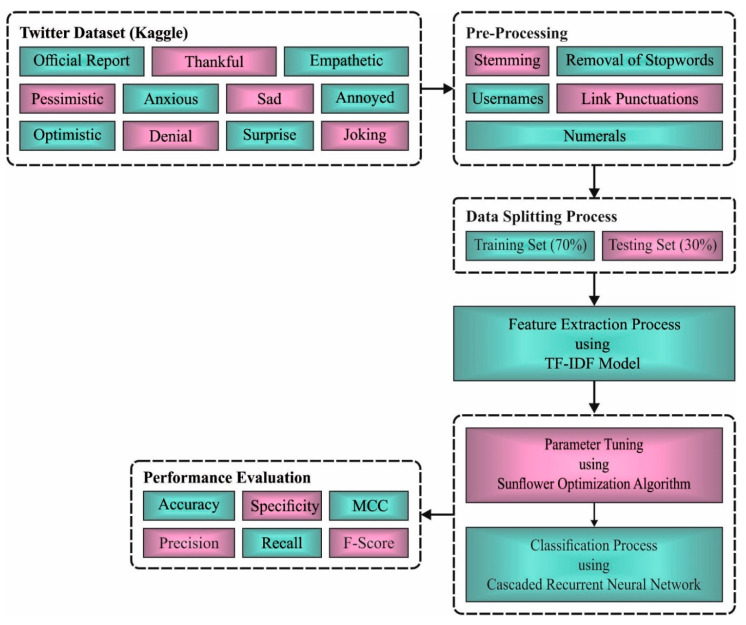
Overall process of SFODLD-SAC technique.

In this study, a novel SFODLD-SAC model was developed for the identification and classification of sentiments on COVID-19 tweets. The presented SFODLD-SAC model follows a series of processes—namely, preprocessing, TF-IDF feature extraction, CRNN classification, and SFO-based parameter optimization. Figure 1 illustrates the pipeline of the SFODLD-SAC model. The workflow of each module in the SFODLD-SAC model is elaborated in the following subsections.

### 3.1. Data Used

In this section, the performance of the SFODLD-SAC model on the COVID-19 tweet dataset is investigated [25]. The dataset holds 2750 instances with 11 class labels. The details related to the dataset are given in Table 1. Some sample tweets related to COVID-19 are provided in Table 2.

### 3.2. Data Preprocessing

At first, the SFODLD-SAC model preprocessed the tweets in distinct ways such as stemming, removal of stopwords, usernames, link punctuations, and numerals [25].

- Removing usernames and links in tweets that do not affect SA;
- Removing punctuation marks such as hashtags and converting them to lower case;
- Removing stopwords and numerals.

In addition, stemming was performed to reduce the terms to their root forms. The process of reducing the term also aids to reduce the complexity of text features. Then, the TextBlob approach was used to determine the sentiment scores. Afterward, the TF-IDF model was executed to generate a collection of feature vectors. In this study, the TF-IDF model was applied for the useful extraction of features from the preprocessed data.

### 3.3. Sentiment Classification Using CRNN Model

For the effective recognition and classification of sentiments, the CRNN model was exploited [26]. RNN is a branch of an artificial neural network (ANN), that is, a feedforward neural network (FFNN) with connections and loops. Unlike FFNN, RNN is able to calculate input sequence using a recurrent hidden layer with the activation of previous steps. Given the sequential dataset $\left(x_{1},\;x_{2},\;\ldots ,\;x_{T}\right)$, where $x_{i}\;$ denotes the data in $i^{th}$ time step, RNN upgrades the recurrent hidden layer $h_{t}$ as follows:

(1) $$h_{t}=\left\{\begin{array}{ll} 0, & if\;\;\;\;\;t=0.\\ \phi \left(h_{t-1},\;x_{t}\right), & otherwise. \end{array}\right.\;$$

where ϕ indicates a nonlinear function. Therefore, RNN is made up of output $\left(y_{1},\;y_{2},\;\ldots ,\;y_{T}\right)$. Eventually, data classification is implemented by an output $y_{T}$. In the traditional RNN model, the update rule of the recurrent hidden layer in (1) can be implemented by

(2) $$h_{t}=\phi \left(Wx_{t}+Uh_{t-1}\right),$$

where W and U represent the coefficient matrix for input and activation of recurrent hidden units. Given that $p\left(x_{1},\;x_{2},\;\ldots ,\;x_{T}\right)$ is a sequential probability as follows:

(3) $$p\left(x_{1},\;x_{2},\;\ldots ,\;x_{T}\right)=p\left(x_{1}\right)\cdots p\left(x_{T}|x_{1},\;\ldots ,\;x_{T-1}\right).$$

**Figure 2:**
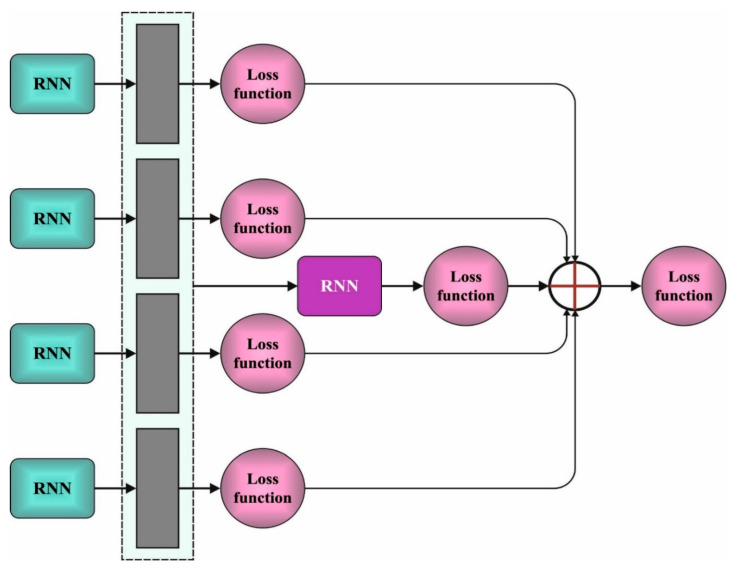
CRNN structure.

Next, the conditional likelihood distribution can be developed by utilizing a recurrent network. The tweets can be processed as sequence data, and a recurrent network is employed to model spectral sequence [26]. In contrast to the LSTM unit, GRU needs a smaller number of variables pertinent for classification, and a fewer number of training instances is needed. Therefore, GRU was chosen as a key element of RNN. The essential component of GRU is 2 gating units that are used to control the data flow within the unit. Figure 2 depicts the framework of CRNN.

(4) $$p\left(x_{t}|x_{1},\;\ldots ,\;x_{t-1}\right)=\phi \left(h_{t}\right),$$

(5) $$h_{t}=\left(1-u_{t}\right)h_{t-1}+u_{t}{\tilde{h}}_{t}.$$

Now, $u_{t}$ symbolizes the update gate as follows:

(6) $$u_{t}=\sigma \left(w_{u}x_{t}+v_{u}h_{t-1}\right).$$

### 3.4. Parameter Optimization

Finally, the SFO algorithm was utilized to optimally adjust the hyperparameters involved in the CRNN model. Gomes et al. [27] introduced an approach for flowering plants based on a flower pollination technique that takes into account the biological process of reproduction.

**Figure 3:**
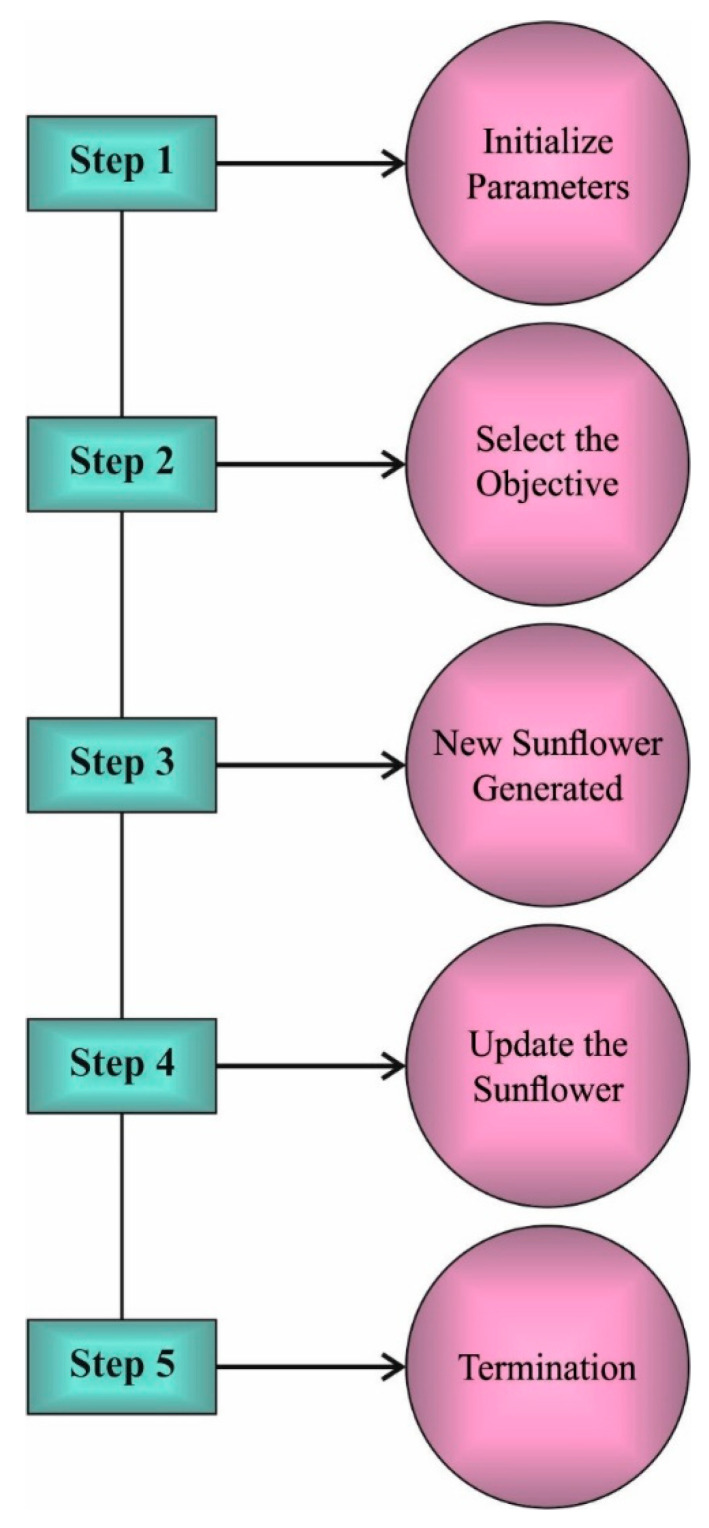
Flowchart of SFO.

Generally, the SFO algorithm involves six steps, as given in Figure 3. It starts with the parameter initiation process, during which the number of sunflowers, maximum iterations, and solution dimension space are initialized. Then, the sunflower parameters such as pollination rate, mortality rate, and survival rate are fixed. In the third step, the optimal objective of every sunflower is arbitrarily chosen. Next, the optimal sunflower is updated. Afterward, the new sunflower is produced depending upon the pollination and mortality rate. In the final step, the termination condition is checked, and the process continues until the stopping criteria are fulfilled. The mathematical modeling of the SFO algorithm is given in what follows.

For this algorithm, we considered the peculiar nature of sunflowers in detecting the optimal direction toward the sun. Pollination was considered to occur randomly, with minimal distance between flower i and flower i+1. Then, the flower patch releases billions of pollen gametes. For simplicity, it was assumed that each sunflower only generates 1 pollen gamete and reproduces individually. Next, the amount of heat Q accomplished by the plant is given by

(7) $$Q_{i}=\frac{P}{4\pi r_{i}^{2}},$$

where P denotes source power, and $r_{i}$ indicates distance amongst current plant and optimal i. The sunflower’s direction toward the sun can be represented as follows:

(8) $$\vec{s_{i}}=\frac{X^{*}-X_{i}}{\left|\left|X^{*}-X_{i}\right|\right|},i=1,\;2,\;\ldots ,\;n_{p}.$$

The sunflowers in direction s are evaluated by

(9) $$d_{i}=\lambda \times P_{i}\left(\left|\left|X_{i}+X_{i-1}\right|\right|\right)\times \left|\left|X_{i}+X_{i-1}\right|\right|,$$

where λ represents constant value, $P_{i}\left(\left|\left|X_{i}+X_{i-1}\right|\right|\right)$ denotes pollination possibility, i.e., sunflower i pollinated with neighboring i−1, creating an individual in an arbitrary position that varies according to the distance among the flowers. Specifically, the individual near the sun would take small steps in the local refinement search. Additionally, it is necessary to bound maximal steps given by the individual. Hence, it is defined as

(10) $$d_{\max}=\frac{\left|\left|X_{\max}-X_{\min}\right|\right|}{2\times N_{\mathrm{pop}}},$$

where $X_{\max}$ and $X_{\min}$ indicates lower and upper bounds, and $N_{\mathrm{pop}}$ represents the number of plants in the population. It can be expressed as follows:

(11) $${\vec{X}}_{t+1}={\vec{X}}_{i}+d_{i}\times \vec{s_{i}}.$$

The SFO approach resolves an FF for achieving enhanced classification performance. In this case, the minimized classifier error rate was assumed to be the FF determined by Equation (12). The best result includes a minimal error rate, and the worse result gains a high error rate.

(12) $$ClassifierErrorRate\left(x_{i}\right)=\frac{number\;of\;misclassified\;tweets}{Total\;number\;of\;tweets}*100.$$

## 4. Performance Validation

### 4.1. Result Analysis

**Figure 4:**
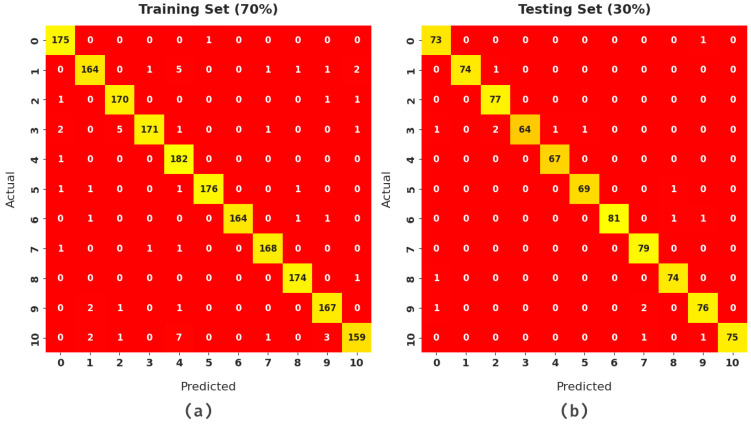
Confusion matrix of SFODLD-SAC technique. (**a**) The training set (TRS) and (**b**) the testing set (TSS).

Figure 4 illustrates a set of confusion matrices formed by the SFODLD-SAC model on a test dataset. The figures indicate that the SFODLD-SAC model ensured the effective identification of distinct class labels on 70% of the training set (TRS) and 30% of the testing set (TSS).

Table 3 provides the detailed classification outcomes of the SFODLD-SAC model on 70% of TRS. The experimental results revealed that the proposed model provided effective outcomes under all class labels.

**Figure 5:**
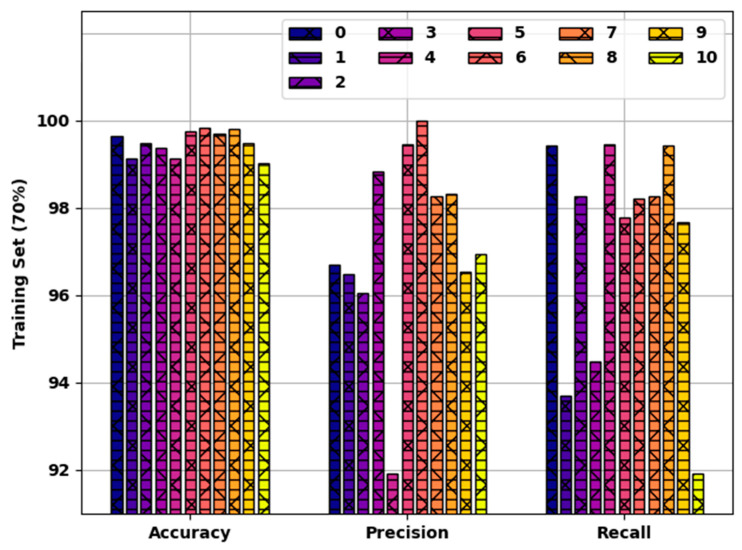
$Acc_{y}$, $Prec_{n}$, and $reca_{l}$ analysis of SFODLD-SAC technique on 70% of TRS for classes 0–10.

Figure 5 reports a brief result of the SFODLD-SAC model on 70% of TRS in terms of $accu_{y}$, $prec_{n}$, and $reca_{l}$. The results indicated that the SFODLD-SAC model accomplished effective results under each class. For instance, the SFODLD-SAC model identified class 0 with $accu_{y}$, $prec_{n}$, and $reca_{l}$ of 99.64, 99.69, and 99.43% correspondingly. In line with this, the SFODLD-SAC model identified class 5 with $accu_{y}$, $prec_{n}$, and $reca_{l}$ of 99.74, 99.44, and 97.78%, respectively. Moreover, the SFODLD-SAC model identified class 10 with $accu_{y}$, $prec_{n}$, and $reca_{l}$ of 99.01, 96.95, and 91.91%, respectively.

**Figure 6:**
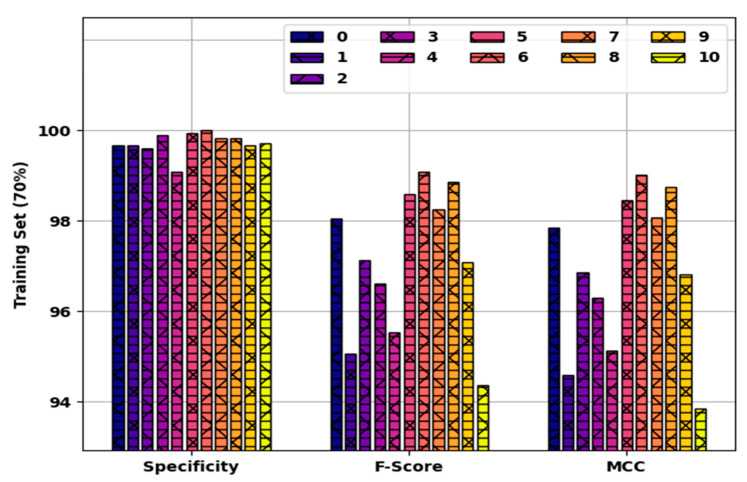
$Spec_{y}$, $F_{score}$, and MCC analysis of SFODLD-SAC technique on 70% of TRS for classes 0–10.

Figure 6 offers detailed results of the SFODLD-SAC model on 70% of TRS in terms of $spec_{y}$, $F_{score}$, and MCC. The experimental values denoted that the SFODLD-SAC model led to proficient performance levels in all classes. For instance, the SFODLD-SAC model recognized class 0 with $spec_{y}$, $F_{score}$, and MCC of 99.66, 98.04, and 97.85%, respectively. In line with this, the SFODLD-SAC model acknowledged class 5 with $spec_{y}$, $F_{score}$, and MCC of 99.94, 98.60, and 98.46%, respectively. In addition, the SFODLD-SAC model categorized class 10 with $spec_{y}$, $F_{score}$, and MCC of 99.71, 94.36, and 93.86%, respectively.

**Figure 7:**
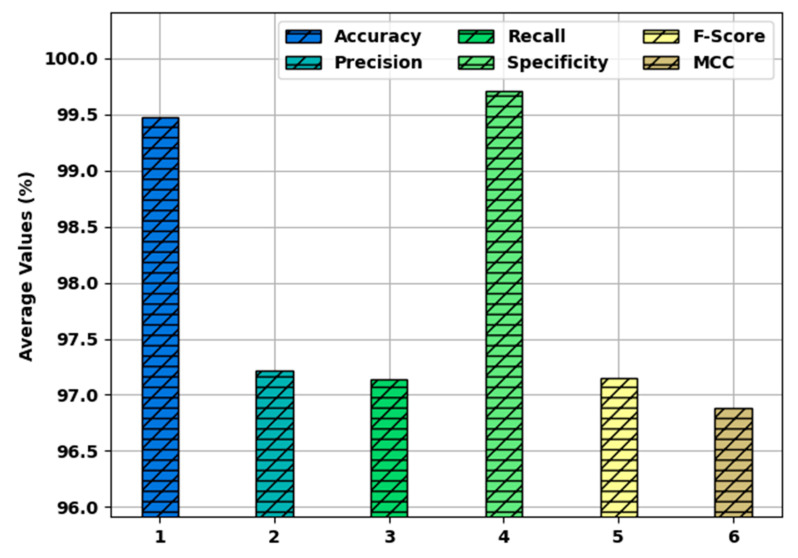
Average analysis of SFODLD-SAC technique on 70% of TRS.

Figure 7 highlights the average classification performance of the SFODLD-SAC model on 70% of TRS. The results indicated that the SFODLD-SAC model accomplished an average $\;accu_{y}$, $prec_{n}$, and $reca_{l}$ of 99.48, 97.22, and 97.14%, respectively. Thus, the SFODLD-SAC model accomplished effective sentiment classification on tweets.

**Figure 8:**
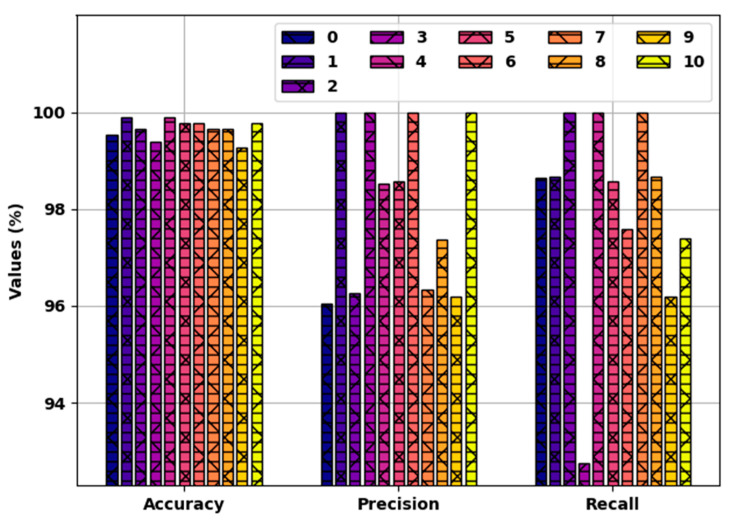
$Acc_{y}$, $Prec_{n}$, and $reca_{l}$ analyses of SFODLD-SAC technique on 370% of TSS for classes 0–10.

Table 4 provides the detailed classification outcomes of the SFODLD-SAC model on 30% of TSS. Figure 8 showcases a comparative result of the SFODLD-SAC model on 30% of TSS in terms of $accu_{y}$, $prec_{n}$, and $reca_{l}$. The figure exhibits that the SFODLD-SAC technique attained improved performance under all class labels. For instance, the SFODLD-SAC model recognized class 0 with $accu_{y}$, $prec_{n}$, and $reca_{l}$ of 99.52, 96.05, and 98.65%, respectively. Moreover, the SFODLD-SAC method identified class 5 with $accu_{y}$, $prec_{n}$, and $reca_{l}$ of 99.76, 98.57, and 98.57%, respectively. Furthermore, the SFODLD-SAC model recognized class 10 with $accu_{y}$, $prec_{n}$, and $reca_{l}$ of 99.76, 100, and 97.40%, correspondingly.

**Figure 9:**
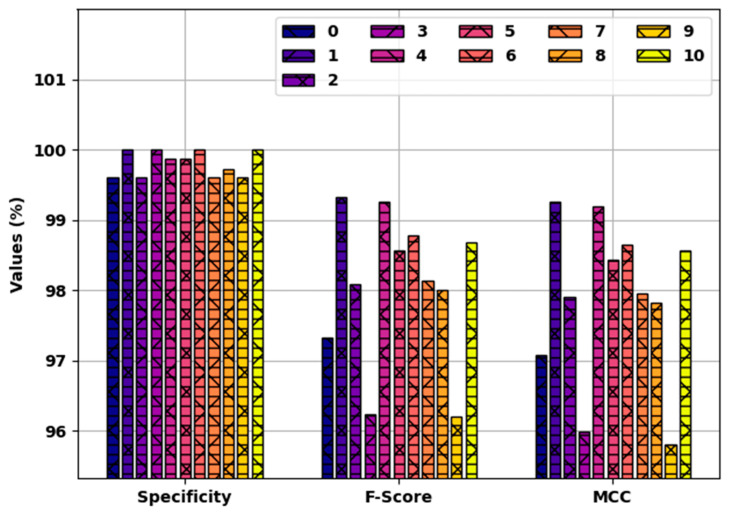
$Spec_{y}$, $F_{score}$, and MCC analyses of SFODLD-SAC technique on 30% of TSS for classes 0–10.

Figure 9 validates a detailed comparative study of the SFODLD-SAC model on 30% of TSS in terms of $spec_{y}$, $F_{score}$, and MCC. The experimental values revealed that the SFODLD-SAC model gained better results under each class. For instance, the SFODLD-SAC model identified class 0 with $spec_{y}$, $F_{score}$, and MCC of 99.60, 97.33, and 97.08%, respectively. At the same time, the SFODLD-SAC model identified class 5 with $spec_{y}$, $F_{score}$, and MCC of 99.87, 98.57, and 98.44%, respectively. Al, the SFODLD-SAC model identified class 10 with $spec_{y}$, $F_{score}$, and MCC of 100, 98.68, and 98.56%, correspondingly.

**Figure 10:**
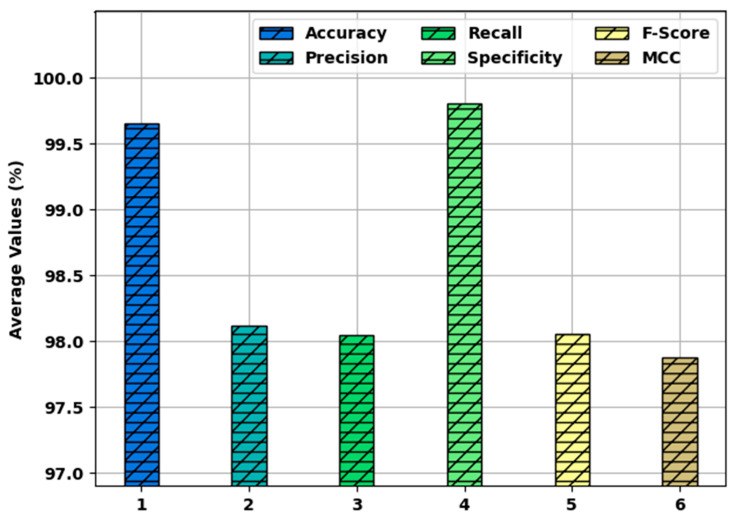
Average analysis of SFODLD-SAC technique on 30% of TSS.

Figure 10 showcases the average classification performance of the SFODLD-SAC model on 30% of TSS. The results revealed that the SFODLD-SAC model provided an average $\;accu_{y}$, $prec_{n}$, and $reca_{l}$ values of 99.76, 98.12, and 98.05%, respectively. Therefore, the SFODLD-SAC model accomplished effective sentiment classification on tweets.

**Figure 11:**
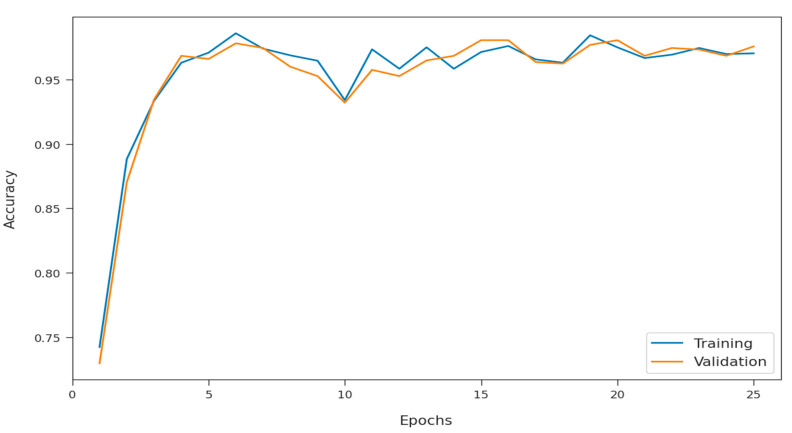
TA and VA analyses of SFODLD-SAC technique.

The training accuracy (TA) and validation accuracy (VA) attained by the SFODLD-SAC model on phishing email classification is demonstrated in Figure 11. Based on the experimental outcomes, the SFODLD-SAC model gained maximum values of TA and VA. Specifically, VA seemed to be higher than TA.

**Figure 12:**
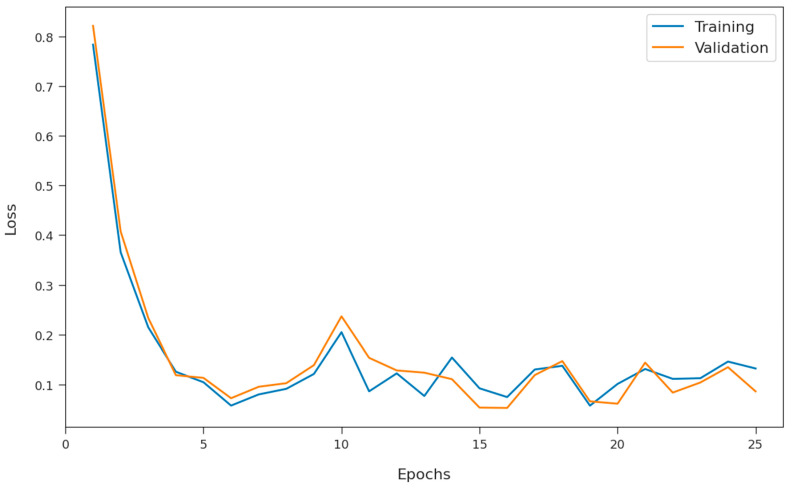
TL and VL analyses of SFODLD-SAC technique.

The training loss (TL) and validation loss (VL) achieved by the SFODLD-SAC model on phishing email classification are shown in Figure 12. Based on the experimental outcomes, it can be inferred that the SFODLD-SAC model accomplished the least values of TL and VL. Specifically, VL seemed to be lower than TL. The results denoted that the SFODLD-SAC model exhibited its ability in categorizing different classes on the test datasets.

### 4.2. Discussion

**Figure 13:**
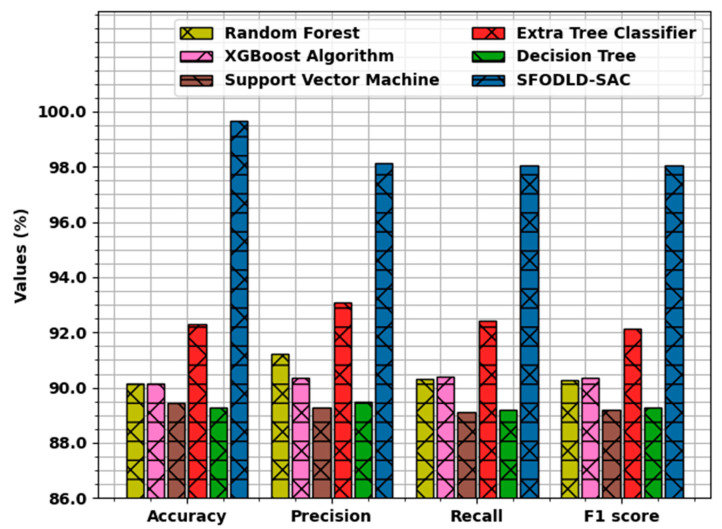
Comparative analysis of SFODLD-SAC technique with existing approaches.

To highlight the supremacy of the SFODLD-SAC model, a comparative study with recent approaches [12] was conducted, the results of which are shown in Table 5 and Figure 13. The experimental outcomes stated that the SVM and DT models showed the least classification performance over the other methods. At the same time, the RF and XGBoost models accomplished slightly improved outcomes over the other techniques. In addition, the extra tree classifier accomplished reasonable performance with $accu_{y}$, $prec_{n}$, $reca_{l}$, and $F1_{score}$ of 92.32, 93.08, 92.42, and 92.13%, respectively.

However, the SFODLD-SAC model accomplished superior outcomes with maximum $\;accu_{y}$, $prec_{n}$, $reca_{l}$, and $F1_{score}$ of 99.65, 98.12, 98.05, and 98.06%, respectively. The above-mentioned results and discussion demonstrate that the SFODLD-SAC model accomplished effective classification performance on COVID-19 tweets. The enhanced performance of the proposed model is due to the optimal hyperparameter tuning of the CRNN model using the SFO algorithm.

## 5. Conclusions

In this study, a novel SFODLD-SAC model was introduced for the recognition and classification of sentiments on COVID-19 tweets. At the initial stage, the SFODLD-SAC model preprocessed the tweets in distinct ways, such as stemming, removal of stopwords, usernames, link punctuations, and numerals. Then, the TF-IDF model was applied for the useful extraction of features from the preprocessed data. Afterward, features were passed into the CRNN model to analyze and classify sentiments. Lastly, the SFO algorithm was utilized to optimally adjust the hyperparameters that exist in the CRNN model. A simulation analysis of the SFODLD-SAC model was performed using a benchmark dataset from the Kaggle repository. Extensive comparative results report the promising performance of the SFODLD-SAC model over other recent state-of-the-art models, with maximum $\;accu_{y}$, $prec_{n}$, $reca_{l}$, and $F1_{score}$ of 99.65, 98.12, 98.05, and 98.06%, respectively. Thus, the presented SFODLD-SAC model can be applied for enhanced SA on COVID-19 tweets, as well as on big data environments to analyze the sentiments in a real-time environment. In the future, outlier detection and clustering models can be employed to improve the sentiment classification performance. Moreover, the proposed SFODLD-SAC model can be extended to the design of an ensemble voting-based fusion model to improve classification performance. In addition, the proposed model can focus on the design of metaheuristic feature selection techniques to reduce the curse of dimensionality. Finally, different data preprocessing approaches can be employed for improving the input data quality in the future.

## Figures and Tables

**Table 1:** Dataset details.

Class Label	Class Name	No. of Instances
Class 0	Optimistic	250
Class 1	Thankful	250
Class 2	Empathetic	250
Class 3	Pessimistic	250
Class 4	Anxious	250
Class 5	Sad	250
Class 6	Annoyed	250
Class 7	Denial	250
Class 8	Surprise	250
Class 9	Official report	250
Class 10	Joking	250

**Table 2:** Sample tweets.

ID	Tweets	Labels
1	NO JOKE I WILL HOP ON A PLANE RN! (Well after COVID-19 lol)	(0) (10)
2	Has anyone else FB ads been killing it since this coronavirus hit?	(0) (5) (10)
3	Im waiting for someone to say to me that all this corona thing is just an April fool’s joke	(3) (4)
4	He is a liar. Proven day night. Time again. Lies when the truth will do. COVID-19	(6)
5	NEW: U.S. CoronaVirus death toll reaches 4000 after nearly 900 new deaths were reported today (BNO News) COVID-19 CoronaVirusOutbreak	(8)
6	Coronavirus impact Govt extends I-T deadlines related to Sections 80C, 80D	(5) (8)
7	That moment you realize your new medication has side effects identical to coronavirus symptoms how will I know?	(4) (9)
8	Watch the government play off Corona virus as a big April Fool’s Joke	(10)
9	The problem of poverty has now covered the cover of religion. The issue has changed. There is relief from corona. All is well	(0) (4)
10	My mental health hasn’t suffered at all under the coronavirus quarantine! Ha-ha, April Fools.	(10)
11	i cannot die before watching a concert live coronavirus pls try to understand	(5) (10)

**Table 3:** Result analysis of SFODLD-SAC technique with distinct measures on 70% of TRS.

Training Set (70%)						
Class Labels	Accuracy	Precision	Recall	Specificity	F-Score	MCC
0	99.64	96.69	99.43	99.66	98.04	97.85
1	99.12	96.47	93.71	99.66	95.07	94.60
2	99.48	96.05	98.27	99.60	97.14	96.86
3	99.38	98.84	94.48	99.89	96.61	96.30
4	99.12	91.92	99.45	99.08	95.54	95.14
5	99.74	99.44	97.78	99.94	98.60	98.46
6	99.84	100.00	98.20	100.00	99.09	99.01
7	99.69	98.25	98.25	99.83	98.25	98.07
8	99.79	98.31	99.43	99.83	98.86	98.75
9	99.48	96.53	97.66	99.66	97.09	96.81
10	99.01	96.95	91.91	99.71	94.36	93.86
**Average**	99.48	97.22	97.14	99.71	97.15	96.88

**Table 4:** Result analysis of SFODLD-SAC technique with distinct measures on 30% of TSS.

Testing Set (30%)						
Class Labels	Accuracy	Precision	Recall	Specificity	F-Score	MCC
0	99.52	96.05	98.65	99.60	97.33	97.08
1	99.88	100.00	98.67	100.00	99.33	99.26
2	99.64	96.25	100.00	99.60	98.09	97.91
3	99.39	100.00	92.75	100.00	96.24	95.99
4	99.88	98.53	100.00	99.87	99.26	99.20
5	99.76	98.57	98.57	99.87	98.57	98.44
6	99.76	100.00	97.59	100.00	98.78	98.65
7	99.64	96.34	100.00	99.60	98.14	97.96
8	99.64	97.37	98.67	99.73	98.01	97.82
9	99.27	96.20	96.20	99.60	96.20	95.80
10	99.76	100.00	97.40	100.00	98.68	98.56
**Average**	99.65	98.12	98.05	99.81	98.06	97.88

**Table 5:** Comparative analysis of SFODLD-SAC technique with existing approaches.

Methods	Accuracy	Precision	Recall	F1 Score
Random Forest	90.13	91.22	90.30	90.29
XGBoost Algorithm	90.16	90.35	90.39	90.36
Support Vector Machine	89.43	89.29	89.12	89.18
Extra Tree Classifier	92.32	93.08	92.42	92.13
Decision Tree	89.29	89.47	89.21	89.29
SFODLD-SAC	99.65	98.12	98.05	98.06

## Data Availability

Data sharing is not applicable to this article, as no datasets were generated during the current study.
